# A Nuisance That Ought to Be Abated; Education of Feeble-Minded Children

**Published:** 1873-08-01

**Authors:** 


MEDICAL EXAMINER.

*A Semi-Monthly Journal of Medical Sciences.*

EDITED BY

N. S. DAVIS, M. D., AND F. H. DAVIS, M. D.

Chicago, August 1st, 1873.

### EDITORIAL.

### A NUISANCE THAT OUGHT TO BE ABATED.

For many years a class of unprincipled and reckless public impostors have been in the habit of using the names of prominent members of the medical profession to aid them in deceiving the community.

Every few months we receive a hand-bill, issued by some traveling mountebank, claiming extraordinary skill in the treatment of something, with the name of "Prof. N. S. Davis, of Chicago," as a reference. Recently we saw in a daily newspaper, published somewhere in Kentucky, an advertisement of " . . . . Bloom of Youth," a cosmetic for ladies. In the advertisement was a paragraph stating that "Dr. Louis A. Sayre, one of the most eminent physicians of New York, had examined its ingredients, and pronounced it entirely harmless." It attracted our attention simply from the fact that only four years since Dr. Sayre read to the American Medical Association several cases of very aggravated paralysis, occasioned by the free use of this very cosmetic, and showed that it contained both acetate and carbonate of lead in its composition. Yet these infamous public impostors boldly publish him as directly endorsing the harmlessness of their stuff. There ought to be some legal method of inflicting speedy and severe punishment on such offenders.

### EDUCATION OF FEEBLE-MINDED CHILDREN.

An institution for this purpose was established at Jacksonville, Illinois, in 1865, and has been doing a good work for that unfortunate class of children ever since. The last Legislature adopted the institution, and placed it on the list of State charities. It is under the excellent superintendence of Dr. C. T. Wilbur, of Jacksonville, who is desirous of obtaining the names and residences of all the feeble-minded children in the State. The next term of the school commences early in September. For all further needed information, address Dr. C. T. Wilbur, Superintendent, Jacksonville, Illinois.

